# Real-world impact of nirsevimab immunisation and maternal RSV vaccination against respiratory disease on emergency department attendances and admissions: a multinational retrospective analysis

**DOI:** 10.1016/j.lanepe.2026.101597

**Published:** 2026-02-03

**Authors:** Aida Perramon-Malavez, Antonio Chiaretti, Ermengol Coma, Mihai Craiu, Steve Foster, Paul Leonard, Robin Marlow, Valtýr Thors, Montserrat Martínez-Marcos, Jacobo Mendioroz, Jorgina Vila, Anna Creus-Costa, Clara Prats, Damian Roland, Antoni Soriano-Arandes, Thomas C. Williams, Danilo Buonsenso

**Affiliations:** a Computational Biology and Complex Systems (BIOCOM-SC) Group, Department of Physics, Universitat Politècnica de Catalunya (UPC), Catalonia, Spain; b Department of Woman and Child Health and Public Health, Fondazione Policlinico Universitario A. Gemelli IRCCS, Rome, Italy; c Area Pediatrica, Dipartimento di Scienze della Vita e Sanità Pubblica, Università Cattolica del Sacro Cuore, Roma, Italy; d Primary Care Services Information System (SISAP), Institut Català de la Salut (ICS), Barcelona, Catalonia, Spain; e Carol Davila University of Medicine and Pharmacy, 050474, Bucharest, Romania; f National Institute for Mother and Child Health, Alessandrescu-Rusescu, 020395, Bucharest, Romania; g Emergency Department, Royal Hospital for Children, Glasgow, UK; h Emergency Department, Royal Hospital for Children and Young People, Edinburgh, UK; i Emergency Department, Bristol Royal Hospital for Children, Bristol, UK; j Children's Hospital, Reykjavík, Iceland; k Public Health Secretariat, Department of Health, Government of Catalonia, Barcelona, Catalonia, Spain; l Paediatric Hospitalisation Unit, Children's Hospital Vall d'Hebron, Barcelona, Catalonia, Spain; m Infection and Immunity in Paediatric Patients, Vall d’Hebron Research Institute, Barcelona, Catalonia, Spain; n SAPPHIRE Group, Population Health Sciences, Leicester University, Leicester, UK; o Paediatric Emergency Medicine Leicester Academic (PEMLA) Group, Children's Emergency Department, Leicester Royal Infirmary, Leicester, UK; p Department of Paediatrics, Serveis de Salut Integrats del Baix Empordà, Palamós, Girona, Catalonia, Spain; q Child Life and Health, University of Edinburgh, Edinburgh, UK

**Keywords:** Nirsevimab, Maternal vaccination, Bronchiolitis, Lower respiratory tract infections, Children

## Abstract

**Background:**

Nirsevimab, a long-acting monoclonal antibody against respiratory syncytial virus (RSV), was recently introduced in Catalonia (Spain, 2023–2024 season onwards) and Italy (2024–2025 season). The United Kingdom (UK) instead introduced maternal RSV vaccination (RSVpreF) in the 2024–2025 season. Our aim was to analyse emergency department (ED) attendances and admissions to hospital following RSVpreF and nirsevimab introduction, with hospitals in Iceland and Romania, where no intervention was introduced, used as comparators.

**Methods:**

Multi-national retrospective analysis of ED attendances and admissions for all diagnoses, respiratory diagnoses excluding bronchiolitis, and bronchiolitis from all hospitals in Catalonia (Spain), four UK hospitals (Bristol, Edinburgh, Glasgow, and Leicester), and one hospital in Italy (Rome), Romania (Bucharest), and Iceland (Reykjavík) from April 2018 to March 2025. Bronchiolitis diagnoses in the 2024–2025 season were compared to previous pre-intervention seasons (2018–2023, excluding the 2020–2021 COVID-19 year) by applying a generalised linear model in Poisson regression to obtain risk ratios (RR) and 95% confidence intervals (95% CI).

**Findings:**

In the 2024–2025 season, in Catalonia, there was a reduction in the RR for bronchiolitis ED attendances and admissions in infants of age <6 months (RR 0.45; 95% CI 0.43–0.47 and RR 0.40; 95% CI 0.37–0.43, respectively). This was not seen in Rome, where the RR for ED attendances with bronchiolitis in infants of age <6 months was 1.09 (95% CI 0.92–1.30) and the RR for admissions was 1.12 (95% CI 0.83–1.52). In the UK, for infants of age <6 months with bronchiolitis, there was a significant but modest reduction in 1 out of 4 hospitals for ED attendances (Leicester; RR 0.91, 95% CI 0.85–0.97) and in 2 out of 4 hospitals for admissions (Leicester; RR 0.80, 95% CI 0.69–0.94 and Edinburgh; RR 0.85, 95% CI 0.76–0.95).

**Interpretation:**

In Catalonia, there was a sustained reduction in bronchiolitis ED attendances and admissions for infants in the 2024–2025 season. However, no substantial reduction in bronchiolitis ED attendances or admissions was observed in Rome or the UK. These differences are likely to relate to the reduced uptake of RSV prevention products in these settings compared to Catalonia.

**Funding:**

None.

**Research in context**

**Evidence before this study**

We searched PubMed to identify relevant literature from January 30, 2025 (the end date of the search for our previous analysis) until September 12, 2025, using the key words “nirsevimab”, “RSV bronchiolitis” and “maternal vaccin∗”. We identified 22 studies, of which 4 were original studies looking at RSV burden prior to any intervention, 4 were original studies or reviews looking at nirsevimab and maternal vaccination uptake in eligible populations, and 13 were reviews or economic cost-modelling analyses looking at the introduction of RSV prevention measures in general. One study from the United States examined the impact on hospital admissions following the combined introduction of nirsevimab and maternal vaccination in the 2024–2025 season, showing a reduction in hospital admission rates compared to previous years. However, as both products were introduced at the same time, it was unable to distinguish between the relative effects of each intervention.

**Added value of this study**

This is the first multinational European study examining the impact of different preventive strategies against RSV on respiratory disease in infants attending Emergency Departments (EDs), and hospital admissions. The previously demonstrated impact of nirsevimab on reducing the burden of bronchiolitis ED attendances/admissions for infants aged <12 months was maintained in Catalonia (Spain) in the 2024–2025 season, following its initial introduction in the 2023–2024 season. No impact was seen in the study site in Rome (Italy) in the first season of implementation of nirsevimab. In the UK, 1 out of 4 study sites showed a reduction ED attendances for bronchiolitis, and 2 out of 4 study sites showed a significant, but modest, reduction in hospital admissions for bronchiolitis in the 2024–2025 season. In control sites in Iceland and Romania, where no population RSV preventive measures were introduced, increases were seen in hospital admissions for bronchiolitis in the 2024–2025 season compared to previous years.

**Implications of all the available evidence**

Countries making decisions on preventive measures against RSV should weigh up the costs of implementation approaches against the likely impact of the intervention. The limited impact seen in the UK in the first season of maternal vaccination could be related to low uptake in the first stages of the campaign, and emphasises the value of rolling out a preventive campaign well before the start of the RSV season, and of efforts to increase public awareness of RSV prior to implementation to ensure maximum possible uptake. For nirsevimab, the absence of impact seen at our study site in Rome (Italy), where nirsevimab uptake was around 75%, and only available to infants born from August 2024 to February 2025, in contrast with the results from Catalonia (Spain), where nirsevimab uptake was higher (90–93%), and all infants born from April 2024 onwards were eligible, highlights the need for a coordinated and well-organised approach for the introduction of any RSV preventive approach. Our study provides detailed information about the impact of different RSV preventive measures across different countries, but formal cost-effectiveness analyses are needed to fully inform decision-making. Future research should focus on evaluating the economic implications of these interventions.

## Introduction

Respiratory syncytial virus (RSV) is a common cause of hospitalisation for lower respiratory tract infections (LRTIs) in infants and children in Europe.^1^ Since 2023, two products have been recommended by various National Immunisation Technical Advisory Groups in Europe for the prevention of severe RSV disease in infants: the long-acting monoclonal nirsevimab (Beyfortus®) to be administered to infants in their first year of RSV exposure, and a stabilised prefusion protein RSV vaccine (RSVpreF; Abrysvo®), given to pregnant women to prevent severe disease in infants after birth. Nirsevimab has been available for several European countries, including Spain,^2^ since the autumn of 2023. RSVpreF maternal vaccination was introduced to several locations across Europe (including the United Kingdom^3^ and France^4^ from late summer 2024. Recent studies have demonstrated the effectiveness of both nirsevimab and RSVpreF in a real-world setting, with an estimated effectiveness of 83% for nirsevimab^5^ and 71–79% for RSVpreF^6^^,^^7^ in preventing infant hospitalisation with RSV in a real-world setting. However, there is limited data on the relative impact of these two approaches in reducing the burden of RSV disease, and to what degree the effectiveness of nirsevimab is related to the particular strategies used in different locations. Additionally, there are theoretical concerns that a reduction in RSV disease burden in the first year of life could be associated with a consequent increase in RSV burden in the second year of life, at a population level, as was reported in some countries following the easing of lockdown measures.^8^

In a previous study, we demonstrated the real-world impact of nirsevimab in Catalonia, Spain^9^ in its first season of implementation, with no comparable changes seen in comparison countries with no RSV prevention products, but highlighted how season-to-season variability in RSV Emergency Department (ED) attendances and admissions, and between-centre and between-country differences in attendance coding might complicate the interpretation of the impact of interventions in the future. Modelling studies^10^ have suggested that there would be a significant impact of a maternal RSV vaccination campaign on the burden of infant disease, but real-world evidence of impact is currently lacking. Here, we use longitudinal datasets from three geographical areas (Catalonia (Spain), Italy, and the United Kingdom) with the aim of directly comparing the impact of nirsevimab with maternal vaccination, examine the relative impact of nirsevimab when implemented using different strategies in different geographical locations, and understand whether there is year-to-year variability in the protection offered by nirsevimab. We use data from two new locations (Iceland and Romania) where no population-level RSV prevention products for infants had been introduced, as a control to understand how season-to-season variability in the prevalence of RSV could affect the interpretation of impact data.

## Methods

A STROBE checklist^11^ for the study is available in the Supplementary Appendix. We collected data from the EDs of all the centres in Catalonia (Spain), Gemelli University Hospital in Rome (Italy), the Bristol Royal Hospital for Children in Bristol (UK), the Leicester Royal Infirmary in Leicester (UK), the Royal Hospital for Children, Glasgow (UK) and the Royal Hospital for Children and Young People, Edinburgh (UK), Landspitali University Hospital (Reykjavík, Iceland) and National Institute for Mother and Child Health (Bucharest, Romania). Study centres were selected to compare hospital outcomes in Catalonia, a region where nirsevimab was widely implemented since 2023–2024, with a site that introduced nirsevimab for the first time in the 2024–2025 season (Italy), sites that introduced maternal vaccination for the first time in the 2024–2025 sites (UK), and two sites that were yet to introduce any population-level RSV preventative measures (Iceland and Romania).

### Details on RSV prevention strategies at each study site

Details on RSV prevention approaches for high-risk and non-high-risk infants, including eligibility and estimates of coverage in the 2024–2025 winter season are provided in Table 1.

**Table 1:** RSV prevention approaches across study sites for the 2024–2025 season.

Study site	RSV prevention strategy in 2024–2025 season: high-risk infants	RSV prevention strategy in 2024–2025 season: non-high-risk infants	Estimates of coverage for non-high-risk infants in 2024–2025 season	Availability of prevention strategy
Bucharest (Romania)	Palivizumab for infants born at GA <35 weeks, and those with BPD or CHD	None	N/A	Palivizumab: offered free of charge to all eligible infants Nirsevimab: available with private prescription
Reykjavík (Iceland)	Palivizumab for infants born at GA <28 weeks, and those with BPD, CHD, or severe neurological conditions	None	N/A	Palivizumab: offered free of charge to all eligible infants
Edinburgh (Scotland)	Palivizumab for infants with BPD, CDH or SCID	Universal maternal RSVpreF vaccination from 28 weeks gestation from Aug 12, 2024	Maternal vaccination coverage estimates for births in Jan 2025 for Scotland: 57.2%^12^	RSVpreF: Offered free of charge during pregnancy Palivizumab: offered free of charge to all eligible infants
Glasgow (Scotland)	Palivizumab for infants with BPD, CDH or SCID	Universal maternal RSVpreF vaccination from 28 weeks gestation from Aug 12, 2024	Maternal vaccination coverage estimates for births in Jan 2025 for Scotland: 57.2%^12^	RSVpreF: Offered free of charge during pregnancy Palivizumab: offered free of charge to all eligible infants
Leicester (England)	Palivizumab for infants with BPD, CDH or SCID	Universal maternal RSVpreF vaccination from 28 weeks gestation from Sep 1, 2024	Maternal vaccination coverage estimates for births in Jan 2025 for Midlands: 49.2%^13^	RSVpreF: Offered free of charge during pregnancy Palivizumab: offered free of charge to all eligible infants
Bristol (England)	Palivizumab for infants with BPD, CDH or SCID	Universal maternal RSVpreF vaccination from 28 weeks gestation from Sep 1, 2024	Maternal vaccination coverage estimates for births in Jan 2025 for South West England: 60.5%^13^	RSVpreF: Offered free of charge during pregnancy Palivizumab: offered free of charge to all eligible infants
Rome (Italy)	Prior to Nov 25 2024 those with BPD, CHD or immunodeficiency eligible for palivizumab After Nov 25 2025 those with BPD, CHD or immunodeficiency eligible for nirsevimab until age 23 months	Nirsevimab, given after birth for infants those born from Nov 25, 2024, to Feb 28, 2025, and as catch-up to those born from Aug 17 to Nov 24, 2024	Nirsevimab coverage estimates for Italy as a whole: 75%,^14^ although roll-out challenged by initial uncertainty around funding (see main text)	Nirsevimab: offered free of charge to all infants in Rome after decision by Italian MoH in Oct 2025 Palivizumab: offered free of charge to all eligible infants before nirsevimab roll out
Catalonia (Spain)	As per non-high-risk infants if < 12 months of age. If high risk (BPD, CHD or immunodeficiency) and 12–23 months eligible for 2nd nirsevimab dose entering RSV season	Nirsevimab, given after birth for infants born from Oct 1, 2024, to Mar 31, 2025, and as catch-up for those born from Apr 1 to Sep 30, 2024, at the beginning of the RSV season	Nirsevimab coverage: For infants born during the RSV season: 90.3%^15^ For infants born before RSV season (catch-up cohort): 93.6%^15^	Nirsevimab: Offered free of charge for all eligible children

Abbreviations: GA = Gestational Age, BPD = Bronchopulmonary Dysplasia, CHD = Congenital Heart Disease, N/A = Not applicable, MoH = Ministry of Health.

#### Catalonia, Spain

Catalonia recommended nirsevimab for all infants born from April 2023 onwards as part of the regional infant immunisation program; it was made free of charge for all eligible infants. The recommendations included immunisation for all infants born before the winter season, between April 1 and September 30, at primary care centres (PCCs) during October, as well as for all newborns born during the epidemic season, between October 1 and March 31, in public and private hospitals within the first days of life before hospital discharge. Additionally, nirsevimab was made available throughout the entire epidemic season at PCCs to ensure accessibility for newborns who were not immunised in hospital settings, so that those who did not receive it as a newborn were still able to access it at any point throughout the season. Nirsevimab dosing was as per the European Medicines Agency recommendations: 50 mg for those <5 kg and 100 mg for those ≥5 kg.^16^ Roll-out of nirsevimab was accompanied by a public health campaign to raise awareness of RSV and nirsevimab, with training materials for health professionals, and promotional materials for the general public.^15^ Catalonia's nirsevimab campaign for the season 2024–2025 was the same as used as for 2023–2024, immunising all infants from October 1 2024 until March 31 2025, with a catch-up cohort of infants born between April 1 and September 30 2024.^15^ Uptake in the second season of nirsevimab roll-out remained high, with an estimated 90.3% of infants born in the RSV season receiving this, and 93.6% in the catch-up cohort^15^ [Table 1]

#### Rome, Italy

In Italy nirsevimab roll-out was preceded by a period of uncertainty, with an initial announcement from the Italian Ministry of Health that only some regions of country would receive allocated funding for nirsevimab; Lazio, where Rome is located, was initially not one of these eligible regions. Following a period of controversy,^17^ in October 2024 the AIFA (Italian Medicines Agency for Drugs) announced that nirsevimab would be provided for all regions free of charge by the Italian National Health Service.^14^

In Rome, nirsevimab was administered to newborns during hospitalisation from November 25 h to February 28, whereas children born between August 17 and November 24 could receive the monoclonal antibody in an outpatient setting (family paediatrician or vaccination hubs) as part of a catch-up campaign. Older infants with co-morbidities were eligible for nirsevimab, but delivery to this group was hampered by supply issues, unless they were already receiving palivizumab (in this case they either completed palivizumab or shifted to nirsevimab).

To publicise the roll-out, healthcare professionals received official emails from the national health system, as general paediatricians were involved in administering the drug in ambulatory settings, as well as in vaccination centres. In addition, major national societies like the Italian Society of Paediatrics also advertised its use through mailing lists and official social media channels. The public was informed by neonatologists at time of delivery, family paediatricians and through a social media campaign.^18^

Estimates for coverage across the whole country are that 75% of eligible infants in Italy received nirsevimab by the end of the season.^14^ Nirsevimab dosing was as per the European Medicines Agency recommendations: 50 mg for those <5 kg and 100 mg for those ≥5 kg.^16^

#### Bristol, Edinburgh, Glasgow and Leicester, United Kingdom

In the UK, RSVpreF vaccination was introduced free of charge for all pregnant women at a gestation of 28 weeks or more, from August 12, 2024, in Scotland, and September 1, 2024, in England.^7^ The launch of the vaccination campaign was preceded by public health campaigns to raise awareness amongst both health professionals and the general public.^19^ Uptake of maternal RSVpreF for live births in January 2025 was 57.2% across Scotland,^12^ 49.2% in the Midlands (Leicester) and 60.5% in South-West England (Bristol)^13^ [Table 1].

#### Reykjavík, Iceland

During the study period in Iceland, RSV prophylaxis for high-risk paediatric populations was administered exclusively with palivizumab, which was given free at point of care to all eligible infants. Those eligible were those born at <28 weeks, or those children <2 years of age with a confirmed diagnosis of bronchopulmonary dysplasia, congenital heart disease with significant hemodynamic compromise or severe neuromuscular disability.^20^

#### Bucharest, Romania

During the study period in Romania, government funded RSV prophylaxis for high-risk paediatric populations was provided using palivizumab. Eligible groups comprised (i) preterm infants born at <35 weeks’ gestational age who were <6 months of age at the onset of RSV seasonal circulation, and (ii) children <2 years of age with a confirmed diagnosis of bronchopulmonary dysplasia or congenital heart disease with significant hemodynamic compromise. Palivizumab was fully reimbursed by the national healthcare system for all children meeting established eligibility criteria.^21^ Nirsevimab was authorized by the Romanian National Agency for Medicines and Medical Devices (ANMDMR) on 31 October 2022.^22^ However, in the 2024–2025 season, it was not included in the national reimbursement scheme. Consequently, its clinical use remained extremely limited, being confined to isolated cases in which infants were born or followed up in private healthcare facilities during early infancy.

### Data on ED attendances and hospital admissions

For each site, for seasons April 1, 2018, to March 31, 2019, until April 1, 2024, to March 31, 2025, we gathered total ED attendances and total admissions from the ED for all diagnoses, respiratory diagnoses (RD) excluding bronchiolitis, and bronchiolitis diagnoses for children <6 months, 6–11 months, and 12–23 months. The codes used for these diagnoses (ICD-10 for Catalonia, Rome [Italy], Reykjavík [Iceland], and Bucharest [Romania]; Emergency Care Data Set [ECDS] and SNOMED CT for the UK sites) are provided in Supplementary Table S1. SNOMED CT is a structured clinical vocabulary for use in electronic health records. The coding procedures are mostly comparable, particularly for this study, when we focused the case-research on well-defined conditions (bronchiolitis and respiratory diagnoses).

In Catalonia, data from hospital admissions and discharges were obtained from the Minimum Basic Data Set (CMBD) and hospital ED visits from the CMBD-UR for all public and private hospitals in Catalonia (n = 68). In the UK, data was obtained from the local Emergency Department Information System associated with that hospital. In Rome, data were obtained from the electronic system of the hospital, coding all attendances and admissions to the hospital. In Iceland and Romania, all data were obtained from the hospital electronic information system. For all sites, ethical permissions allowed for the use of aggregate patient data only, and we were therefore unable to report on attendance/admissions using sex, socio-economic status, or race/ethnicity as descriptive variables.

For Catalonia, data on a total of 1,357,286 ED attendances and 279,212 hospital admissions were included, for the four UK sites 470,238 attendances and 87,786 admissions, for Rome (Italy) 25,768 attendances and 4556 admissions, for Reykjavík (Iceland) 49,328 attendances and 3146 admissions, and for Bucharest (Romania) 85,808 attendances and 22,738 admissions (Supplementary Table S3).

### Statistical analyses

We calculated the percentage of bronchiolitis among all diagnoses, and among RDs and visualised the progression over time of the data on bronchiolitis, RDs and all diagnoses by site and age group. With these datasets, we then calculated a generalised linear model (GLM) in Poisson regression per place (this could be either region [Catalonia] or hospital [for other study sites]) and age group (<6 months, 6–11 months, 12–23 months), obtaining the risk ratio (RR) and 95% confidence intervals (CI) of bronchiolitis ED attendances and admissions. We used a Poisson regression model because our data are highly aggregated and stratified by age group, geographical area, and epidemiological season. This level of aggregation substantially reduces random variation and unobserved heterogeneity, which are the main sources of overdispersion in count data, and is consistent with our previous work using the same data structure.^9^ A result of an RR of, for example, 0.40 means that, compared to the reference period, there were 60% fewer ED visits in the groups of interest. This is because an RR of 0.40 corresponds to 40% of the visits compared to the baseline, indicating a 60% reduction.

Comparisons were made between 2024 and 2025 and all seasons defined as commencing on April 1 of one year and ending on March 31 the subsequent year) before any intervention (2018–2019 to 2022–2023), excluding 2020–2021, labelling them as “pre-intervention” and setting them as a reference level. We excluded the 2020–2021 season from our calculations, as we anticipated that low case rates related to lockdown measures introduced in response to the COVID-19 pandemic could skew average ED attendances/admissions for the preceding and subsequent seasons. We performed an analysis excluding the 2023–2024 season for comparability across all sites, but in order to include data from this year also provide a complementary analysis including that season as a pre-intervention reference period in the Supplementary Appendix (Supplementary Tables S4 and S5), again excluding the 2020–2021 season for this analysis. We added as offset term the natural logarithm of total ED attendances/admissions for comparability. This model can be expressed as:

EQ1 $$In\left(E\left[bronchiolitis\right]\right)=\beta_{0}\;+\;\beta_{1}\;\cdot \;season\;+\;In\left(alldiagnoses\right)$$

where:

E [*bronchiolitis*]: expected number of bronchiolitis diagnoses;

β_0_: Intercept;

β_1_: Coefficient related to the difference for the season with respect to the baseline, in this case, the average between seasons;

*season*: Defined as commencing on April 1 of one year and ending on March 31 the subsequent year.

With the exponential of β_1_ in EQ (1), the RR (95% CI) can be calculated. Our analysis used aggregated data, stratified by age group, location, and epidemiological season; there were no missing data in these stratifications. Python v.3.9.20 and R v. 4.4.2 were used for data analysis and visualisation.

Scripts used for these analyses and visualisation are available at https://github.com/aida-pm/Nirsevimab.

### Ethical approvals

This retrospective observational study involving human participants followed the ethical standards of the institutional and national research committee and with the 1964 Helsinki Declaration and its later amendments or comparable ethical standards. Ethical approval was obtained for aggregate data from Catalonia (Vall d'Hebron Research Institute (EOM(AG)006/2024 (6232)) and Fundació IDIAP-Jordi Gol (25/041-EOm) with the approval dates on March 15, 2024 and March 6, 2025) and Rome (Italy; [Comitato Etico Fondazione Policlinico Universitario A. Gemelli IRCCS ID 3497, Prot 0049226/20, 02/10/2020]). In the UK sites data collection was registered under the umbrella of a service evaluation project; under Health Research Authority guidance these projects are exempt from formal ethical approval. In Iceland, ethical approval was obtained from the ethical committee of Landspitali, University Hospital (10-2021). In Romania, ethical approval was obtained from the ethical committee of NIMCH Alessandrescu-Rusescu (08-2025). Consent was not required at any site for these aggregated, population level datasets.

### Role of the funding source

There was no funding source for this study.

## Results

### Overview of study sites

**Fig. 1:**
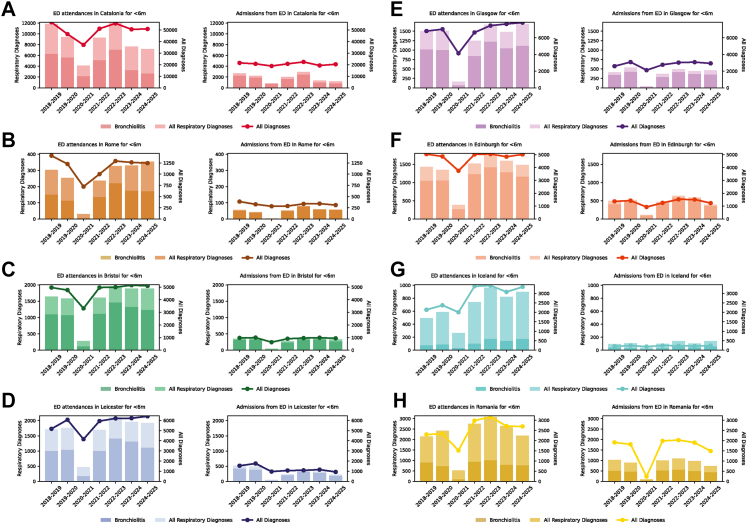
**Emergency Department (ED) attendances and hospitalisations for infants <6 months of age at study sites.** The number of bronchiolitis (dark) and all respiratory diagnoses (light) cases attending each site's ED and admitted from the ED are shown on the left and right of each panel, respectively, with numbers shown on the left y-axis. The right y-axis shows the total number of ED attendances and admissions, respectively, at the study sites. Panels show the sites from the following locations- A: Catalonia; B: Rome; C: Bristol; D: Leicester; E: Glasgow; F: Edinburgh; G: Iceland; H: Romania. For full details of each site please refer to the Methods.

Comparing study sites (Fig. 1) by visual inspection, the number of bronchiolitis ED attendances for infants of age <6 months in Catalonia was lower in both 2023–2024 and 2024–2025 than in previous years, and comparable to the reductions seen in the pandemic year of 2020–2021. However, the number of ED attendances in study sites in other countries showed year-to-year variability, with little evidence of a reduction in bronchiolitis diagnoses in the UK or Rome in the 2024–2025 season compared to previous years.

For hospital admissions with bronchiolitis for infants <6 months of age, again, there was a drop in admissions with bronchiolitis diagnosis in Catalonia in the 2024–2025 season, equal in magnitude what was seen in the 2023–2024 season [8]. In Catalonia, the reduction in bronchiolitis diagnoses did not however appear to result in an overall lower *total attendances* or admissions. The trend for other countries was less clear. Equivalent data for ED attendances and hospital admissions for infants 6–11 months and children 12–23 months of age showed year to year variability for all sites, with no reductions as clear as those seen for bronchiolitis in Catalonia in the 2023–2024 and 2024–2025 seasons and are shown in Supplementary Figures S1 & S2.

### Comparisons of nirsevimab and RSVpreF impacts in different countries

#### Catalonia

**Fig. 2:**
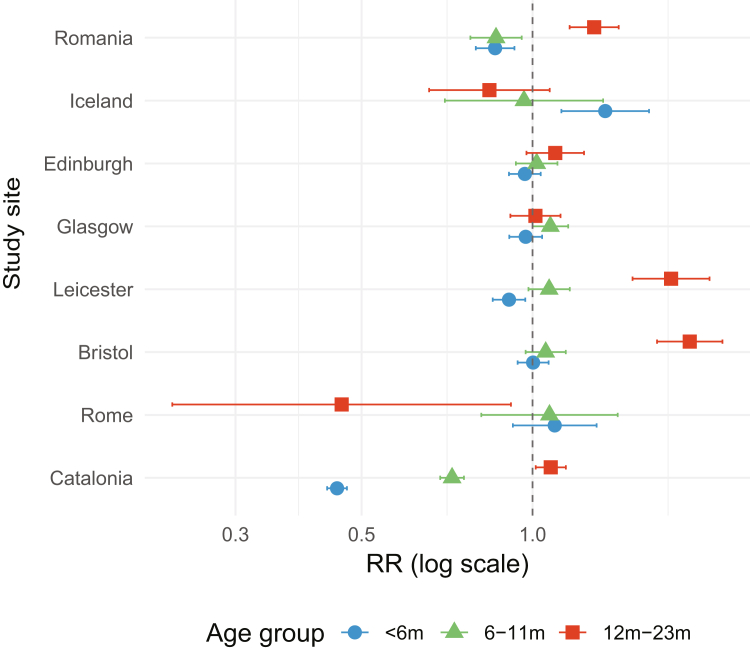
**Impact (risk ratio [RR] with 95% confidence interval) of nirsevimab and RSVpreF on ED attendances with bronchiolitis in the 2024**–**2025 season.** At this point nirsevimab was in place in Catalonia and Rome, RSVpreF maternal vaccination in the United Kingdom, and no RSV population level interventions were in place in Romania and Iceland. RR calculated using years 2018–2023 (excluding the Covid-19 year of 2020–2021) by applying a generalised linear model in Poisson regression.

**Fig. 3:**
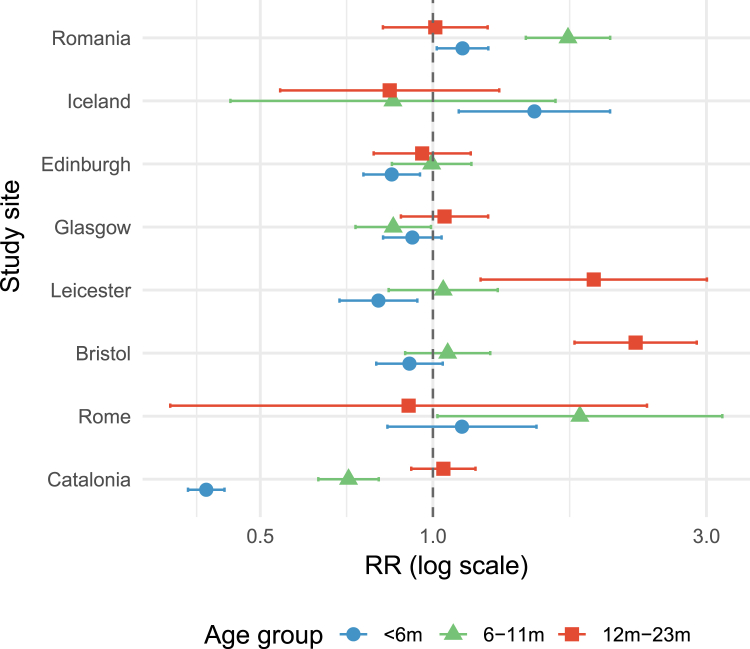
**Impact (risk ratio [RR] with 95% confidence interval) of nirsevimab and RSVpreF on hospital admissions with bronchiolitis in the 2024**–**2025 season.** At this point nirsevimab was in place in Catalonia and Rome, RSVpreF maternal vaccination in the United Kingdom, and no RSV population level interventions were in place in Romania and Iceland. RR calculated using years 2018–2023 (excluding the Covid-19 year of 2020–2021) by applying a generalised linear model in Poisson regression.

Results from the Poisson regression are presented in Fig. 2 (ED attendances) and Fig. 3 (hospital admissions), with Supplementary Tables S4 and S5 giving the values from the regression in more detail. These show a significant reduction in ED attendances and hospital admissions for Catalan infants of age <6 months, in keeping with what was seen in the previous season (2023–2024): the RR for ED attendances in 2024–2025 in infants age <6 months was 0.45 (95% CI 0.43–0.47) compared to previous seasons (excluding 2023–2024) and for attendances was 0.40 (95% CI 0.37–0.43). There was also a reduction in the RR for ED attendances and hospital admissions for infants aged 6–11 months (0.72 [95% CI 0.69–0.76] and 0.71 [95% CI 0.63–0.80], respectively). A very modest increase of 1.08 (95% CI 1.01–1.14) was seen in the RR of ED attendances with bronchiolitis in children of age 12–23 months compared to previous years, but no increase was seen in the RR of hospital admissions for bronchiolitis.

#### Italy

In Rome, no change was seen in ED attendances or admissions for bronchiolitis in infants of <6 months, with a RR for ED attendances of 1.09 (95% CI 0.92–1.30) and admissions of 1.12 (95% CI 0.83–1.52). However there was an increase in hospital admissions in those 6–11 months of age (RR: 1.80 [95% CI 1.02–3.20]) compared to previous years. A significant decrease in ED attendances was observed for 12–23 months old (RR: 0.46 (95% CI 0.23–0.92)), but the sample size in these groups is relatively small, hence the statistical power is decreased.

#### United Kingdom

In the United Kingdom, one site (Leicester) showed a significant reduction of RR 0.91 (95% CI 0.85–0.97) in ED attendances with bronchiolitis for infants of age <6 months in the 2024–2025 season, but this was not seen in any of the other UK sites. A significant reduction was seen in admissions for infants of age <6 months in Leicester and Edinburgh (0.80 [95% CI 0.69–0.94] and 0.85 [95% CI 0.76–0.95], respectively), but no reduction was seen for Bristol or Glasgow.

#### Romania and Iceland

A reduction was seen in ED attendances in Romania for bronchiolitis in infants of age <6 months (RR: 0.86, 95% CI 0.79–0.93) and 6–11 months (RR: 0.86, 95% CI 0.77–0.96) in the 2024–2025 season, despite no new intervention having been introduced; in Iceland an increase in ED attendances in the <6 months age group was seen in the 2024–2025 season with a RR of 1.34 (95% CI 1.12–1.60). For hospital admissions with bronchiolitis, an increase in the RR was seen in Romania for both <6 months and 6–11 months age groups, and in Iceland for the <6 months age group.

### Differences when using data from 2018 to 2024 rather than 2018–2023

Using seasons from 2018 to 2019 through to 2023–2024 as a comparator (as opposed to the interval from 2018 to 2019 through to 2022–2023, as presented above) affected 2 of the results for ED attendances, and 2 of the results for hospital admissions (for full details see Supplementary Tables S4 and S5). For Catalonia, the increase seen in ED attendances in the 12–23 month age group in the 2024–2025 season was no longer significant (RR 1.06, 95% CI 0.99–1.12). For Glasgow, there was an increase in ED attendances in the 6–11 month old age group in the 2024–2025 season (RR 1.08 (95% CI 1.00–1.16). For Rome, the increase in hospital admissions in those of age 6–11 months in the 2024–2025 season was attenuated when seasons through to 2023–2024 were used as a comparator (RR of 1.65 compared to 1.80), and in Glasgow a decrease in hospital admissions in those age 6–11 months seen in the 2024–2025 season was no longer significant.

## Discussion

### Summary and interpretation of findings

In this multi-centre, multi-year study, we found that the initial reduction in ED attendances and admissions seen in Catalonia in the 2023–2024 season associated with the introduction of nirsevimab was sustained in its second season (2024–2025). However, in Rome, despite the introduction of nirsevimab for the 2024–2025 season, no difference was seen in ED attendances/admissions in infants aged <6 months or 6–11 months. In the United Kingdom, despite the introduction of RSVpreF maternal vaccination from August (Scotland) and September (England) 2024, a reduction in bronchiolitis ED attendances in infants <6 months was seen in only 1/4 sites, and a reduction in admissions in 2/4 sites.

The comparison sites of Romania and Iceland showed variable changes in the 2024–2025 season, with Romania seeing a reduction in ED attendances with bronchiolitis in infants aged <6 months compared to previous years, but both sites seeing an increase in bronchiolitis admissions in infants <6 months compared to previous years. These findings highlight the dynamic nature of RSV seasons, and the need to take this into account when interpreting the impact of new campaigns. The more dramatic swings in ED attendances and hospital admissions seen in Romania may relate to local hospital factors: in the Bucharest metropolitan area, four paediatric hospitals provide emergency care, but only two provide major trauma services, not including the site included in this study.^23^ Consequently, respiratory infections represent a larger proportion of emergency visits and admissions to the Bucharest study site.^24^ Additionally, the study site is the only public paediatric facility in the city with dedicated isolation units. Parental preference could therefore play a role in the variability observed, as isolation rooms are perceived as safer and more comfortable for young children, and therefore season-to-season variation in infectious disease dynamics could be magnified by this concentration of infectious disease cases.

The lack of changes in ED attendances/hospital admissions seen in Rome and the UK after the introduction of immunisation strategies is incongruent with the demonstrated real-world effectiveness of both nirsevimab (as seen in Catalonia,^25^^,^^26^ observed impact on ED attendances/hospital admissions in this and our previous work,^9^ and a recently published meta-analysis^5^) and RSVpreF vaccination.^6^^,^^7^ In our study, results in Rome run counter to previous nirsevimab effectiveness evidence reported by the Valle d’Aosta pilot program for the introduction of Nirsevimab and also against a recently published Italian multi-centre study.^27^^,^^28^ In Italy, the lack of impact seen could be due to the reduced uptake of nirsevimab seen in Rome (an estimated 75%, Table 1) compared to Catalonia (90.3% in those born during the RSV season, and 93.6% in the catch-up cohort),^15^ and the fact that Italy provided nirsevimab to infants born after August 17, 2024 only, with no catch-up campaign for infants born before this date. In Italy, a recent decrease in paediatric vaccination coverage has been observed, making the Italian government approve a compulsory vaccination law in 2017, but nirsevimab is not currently part of the Italy's mandatory National Immunisation programme.

In the United Kingdom, the catch-up nature of the initial campaign, meaning that many women were vaccinated too close to delivery to fully benefit newborns from maternal RSV vaccination, means that the full effects may not be seen until the second season of the campaign. In the first stages of the vaccination campaign uptake was lower than what was reached later on, so that in September 2024 only 41% of newborns in Scotland^12^ and 34% of newborns in England^29^ had been beneficiaries of RSVpreF maternal vaccination. However, even a year into the campaign, RSV maternal vaccination coverage remained low (53.7% at the latest estimate in England for births in June 2025^30^ and 55.5% in Scotland for births in August 2025^12^). Coverage in England in 2024–2025 for the well-established maternal pertussis vaccine of 65.6%^31^ suggest that RSV maternal vaccination rates may not rise much higher than this in the immediate future. Finally, the benefits of maternal vaccination are known to wane with time, so that protection for infants of age <3 months is greater than those <6 months,^6^^,^^32^ so that older infants are less protected from RSV disease, an effect which is obviated by the nirsevimab catch-up campaign. Therefore, even with higher coverage the effects seen at a population level in the United Kingdom are unlikely to replicate those seen with nirsevimab, where uptake in Catalonia has remained high (>90%) in the second season.^33^

### Implications of the study

The sustained reduction seen in bronchiolitis attendances and admissions in Catalonia highlights that, after the introduction of nirsevimab with careful planning, a reduction in ED over two seasons was maintained throughout this period, probably associated with a well-implemented immunisation strategy. However, ongoing work is likely to be needed to maintain the current high uptake of this intervention. Additionally, although there was a small increase observed in the risk of bronchiolitis ED attendances for those aged 12–23 months (the first Catalonian nirsevimab cohort) in the 2024–2025 season (RR 1.08; 95% CI 1.01–1.14, no increase was seen in hospital admissions for bronchiolitis in this cohort (see also Supplementary Figure S2). This provides reassurance that there is no evidence for a rebound in cases in the second year of life following nirsevimab administration in the first. This likely relates to the fact that nirsevimab does not appear to prevent RSV infection but rather prevent progression to severe disease.^34^ The data from Rome highlights the need for a coordinated and well-organised approach for nirsevimab introduction, and more work is needed to try and maximise coverage in upcoming seasons. The data from the UK highlights how contingent the impact of maternal vaccination is on overall uptake. Furthermore, a recent meta-analysis highlights the fact that maternal vaccination uptake appears to be generally lower than the uptake seen for nirsevimab.^35^

Countries making decisions on whether to introduce maternal RSVpreF vaccination or a long-acting monoclonal antibody such as nirsevimab or clesrovimab (recently licenced by the FDA)^36^ should therefore weigh up the costs of implementation (taking into account the direct costs of a prevention product, and the cost of programmatic implementation) with the expected uptake and likely impact of the interventions. For low- and middle-income countries considering implementing maternal vaccination, the limited impact seen in the United Kingdom in the first season of implementation at least with regard to ED attendances as we report hereby, emphasise the value of rolling out a campaign well before the start of the RSV season to allow the programme to enter a steady state, and of efforts to increase public awareness of RSV, and the effectiveness of the RSVpreF vaccine, prior to implementation.

### Strengths and limitation of the study

Our study has some limitations. Firstly, it is not a study at a national or regional population level, except for Catalonia. We are comparing hospitals which are referral centres for different population sizes or with different paediatric infrastructures for children attending; the lack of hospital level data for Catalonia means that we were unable to include random effects for hospitals. The referral population for each of them is also not homogeneous. This means that they cannot be taken to be representative for the country as a whole. Secondly, the coding for bronchiolitis and other respiratory diseases can vary widely across the participating sites. To mitigate this, we used the same ICD-10 codes for non-UK sites and SNOMED codes for the UK sites (Supplementary Table S1). However, because we used attendance/admissions codes rather than performing individual linkages to microbiology data, we were unable to evaluate laboratory confirmed RSV outcomes after the introduction of different interventions. Thirdly, we were unable to report detailed sex, ethnicity, or socioeconomic status data for attendances/admissions, as we relied on anonymised aggregate data provided by study sites. Additionally, because we stratified attendances/admissions by the age band 0–5 months, we were unable to look at specific outcomes in infants of age 0–3 months, where one would expect the impact of maternal vaccination to be greater. Fourthly, some of the sites (such as Rome and Reykjavík) had smaller sample sizes than the others, meaning that the confidence intervals for our estimates were wide. Finally, the control sites included in the study are limited to Iceland and Romania. In the future, we aim to extend our network to other European sites to be able to compare the impact of RSV interventions at a pan-European level.

On the other hand, the study has some strengths to highlight. Firstly, it is a multinational European study that assesses the real-world impact of different preventive strategies against RSV. In addition, we included two control sites (Iceland and Romania) to document the dynamic nature of RSV seasons, and the need to take this into account when interpreting data about the impact of new campaigns. Finally, we used harmonised disease definitions to be able to compare impacts across different sites.

### Conclusion

The data from Catalonia, where high nirsevimab uptake was associated with a clear reduction in secondary care burden, demonstrates that a carefully planned nirsevimab strategy can achieve sustained reductions in bronchiolitis ED attendances and admissions, provided that high levels of coverage are maintained. In contrast, data from Rome and the United Kingdom, where uptake of nirsevimab and RSVpreF respectively were lower than Catalonia, and fewer infants were able to benefit, underscore the extent to which the real-world impact of different interventions is contingent upon both organisational factors and the type of strategy adopted. Taken together, these findings indicate that countries considering the introduction of maternal vaccination, or long-acting monoclonal antibodies, should undertake a rigorous assessment of not only the potential effectiveness and real-world impact of each option, but also their relative cost-effectiveness within the local context. Furthermore, these evaluations should be accompanied by concerted international efforts to achieve more equitable pricing, thereby facilitating the adoption of the most effective and sustainable strategy across diverse settings.

## Contributors

ASA, CP, DR, TCW, and DB conceptualised the study. APM, CP, TCW performed statistical analyses. DB, AC, EC, MC, SF, PL, RM, VT, MMM, JM, JV, ACC were responsible for data collection. All authors had access to all data, and ASA and CP verified the data. All authors read and approve the final version of the manuscript and had responsibility for the decision to submit for publication.

## Data sharing statement

The dataset will be held for a minimum of 3 years and is available to be shared on reasonable request to the corresponding author, by providing a formal request supported by a hypothesis, research proposal that included a statistical plan and a local ethic committee approval. A data sharing agreement will need to be signed.

## Declaration of interests

Dr Damian Roland declares grants or contracts from Wellcome Trust (Funding for administrative support for the BronchStop study), Respiratory Syncytial Virus Consortium in Europe (RESCEU) (Funding to establish the BronchStart/Stop study), Imperial College London (Funding for administrative support for the BronchStop study), National Institute for Health Research (Funding for data collection for the study). Dr. Thomas Williams declares funding from the Wellcome Trust: funding for administrative support for the BronchStop study. Dr Antoni Soriano-Arandes declares consulting fees, honoraria for lectures and support for attending meetings from Sanofi, MSD, and Pfizer, and grants or contracts from la Marato tv3 (Funding addressed to my institution Vall d’Hebron Research Institute for a project about respiratory viruses). Clara Prats declares payments or honoraria for lectures by Societat Catalana de Pediatria (Presentation including results of the impact of nirsevimab in Catalonia), grants or contracts by Fundacion BBVA (Funding paid to the university), and Fundacio la Marato tv3. Aida Perramon-Malavez declares grants or contracts from Fundacio la Marato tv3 (Funding addressed to my institution Vall d’Hebron Research Institute for a project about respiratory viruses). Dr Jorgina Vila declares honoraria for presentation and support for attending meetings from Sanofi. Anna Creus-Costa declares support for attending meetings and/or travel by Sanofi and grant VHIR-AULA-2025-016 by Aula Vall d’Hebron. The rest of the authors have nothing to declare.
