# Supplementary material for: Real-world impact of nirsevimab immunisation and maternal RSV vaccination against respiratory disease on emergency department attendances and admissions: a multinational retrospective analysis
Source: Lancet Reg Health Eur. 2026 Feb 3;63:101597. doi: 10.1016/j.lanepe.2026.101597 (PMC12887189; doi:10.1016/j.lanepe.2026.101597)
Supplement: Supplementary Figures and Tables [file mmc1.docx]

**Supplementary Appendix**

**Real-world impact of nirsevimab immunisation and maternal RSV vaccination against respiratory disease in infants: a multi-national retrospective analysis of emergency department attendances and admissions**

[1. Supplementary Tables 2](#_Toc214035387)

[2. Supplementary Figures 12](#_Toc214035388)

[3. STROBE checklist 16](#_Toc214035389)

## 1. Supplementary Tables

**Supplementary Table 1. Study codes.** ICD-10 codes were used for Catalonia, Rome (Italy), Landspitali University Hospital (Reykjavík, Iceland), National Institute for Mother and Child Health (Bucharest, Romania). Emergency Care Data Set (ECDS) and SNOMED CT codes were used for the UK sites.

| **ICD10 mapping** | **ICD10 Description** | **Group** |  |  |
| --- | --- | --- | --- | --- |
| J04.2 | Acute laryngotracheitis | Acute tracheitis |  | Not specifically coded for in ECDS |
| J20.3 | Acute bronchitis due to coxsackievirus | Acute bronchitis, specified |  |  |
| J20.4 | Acute bronchitis due to parainfluenza virus | Acute bronchitis, specified |  |  |
| J20.5 | Acute bronchitis due to respiratory syncytial virus | Acute bronchitis, specified |  |  |
| J20.6 | Acute bronchitis due to rhinovirus | Acute bronchitis, specified |  |  |
| J20.7 | Acute bronchitis due to echovirus | Acute bronchitis, specified |  |  |
| J20.8 | Acute bronchitis due to other specified organisms | Acute bronchitis, specified |  |  |
| J21.8 | Acute bronchiolitis due to other specified organisms | Acute bronchiolitis, specified |  | All coded for under Bronchiolitis (SNOMED 4120002) |
| J21.1 | Acute bronchiolitis due to human metapneumovirus | Acute bronchiolitis, specified |  |  |
| J21 | Acute bronchiolitis | Acute bronchiolitis, specified |  |  |
| J21.0 | Acute bronchiolitis due to respiratory syncytial virus | Acute bronchiolitis, specified |  |  |
| J10 | Influenza due to identified seasonal influenza virus | Influenza |  | All coded for under Influenza (SNOMED 6142004) |
| J10.0 | Influenza with pneumonia, seasonal influenza virus identified | Influenza |  |  |
| J10.1 | Influenza with other respiratory manifestations, seasonal influenza virus identified | Influenza |  |  |
| J10.8 | Influenza with other manifestations, seasonal influenza virus identified | Influenza |  |  |
| J11 | Influenza, virus not identified | Influenza, no identified |  |  |
| J11.0 | Influenza with pneumonia, virus not identified | Influenza, no identified |  |  |
| J11.8 | Influenza with other manifestations, virus not identified | Influenza, no identified |  |  |
| J12.0 | Adenoviral pneumonia | Pneumonia, specified |  | All coded for under Lobar or Bronchopneumonia (SNOMED 233604007 and 278516003 |
| J12.1 | Respiratory syncytial virus pneumonia | Pneumonia, specified |  |  |
| J12.2 | Parainfluenza virus pneumonia | Pneumonia, specified |  |  |
| J12.3 | Human metapneumovirus pneumonia | Pneumonia, specified |  |  |
| J12.8 | Other viral pneumonia | Pneumonia, specified |  |  |
| J18.8 | Other pneumonia, organism unspecified | Pneumonia, unspecified |  |  |
| J45.0 | Predominantly allergic asthma | Asthma |  | All coded for under Asthma (SNOMED 195967001) |
| J45.1 | Nonallergic asthma | Asthma |  |  |
| J45.8 | Mixed asthma | Asthma |  |  |

**Supplementary Table 2. Details of attendances and admissions.** Attendances and admissions by age, study site and diagnosis for Catalonia, Bristol, Leicester, Rome, Glasgow, Edinburgh, Iceland, and Romania. From season 2018-2019 to season 2024-2025, age groups were <6 months, 6-11 months, 12-23 months of age.

| **Region** | **2018-2019** | | | **2019-2020** | | | **2020-2021** | | | **2021-2022** | | | **2022-2023** | | | **2023-2024** | | | **2024-2025** | | |
| --- | --- | --- | --- | --- | --- | --- | --- | --- | --- | --- | --- | --- | --- | --- | --- | --- | --- | --- | --- | --- | --- |
| **Catalonia** | **<6m** | **6-11m** | **12m-23m** | **<6m** | **6-11m** | **12m-23m** | **<6m** | **6-11m** | **12m-23m** | **<6m** | **6-11m** | **12m-23m** | **<6m** | **6-11m** | **12m-23m** | **<6m** | **6-11m** | **12m-23m** | **<6m** | **6-11m** | **12m-23m** |
| **Attendances to ED** |  |  |  |  |  |  |  |  |  |  |  |  |  |  |  |  |  |  |  |  |  |
| All Diagnoses | 56668 | 59562 | 114458 | 46438 | 44728 | 84575 | 37132 | 37509 | 79794 | 51141 | 51396 | 99461 | 55721 | 55185 | 98344 | 45120 | 48639 | 89140 | 50902 | 53209 | 98164 |
| Respiratory Diagnoses (w/o Bronchiolitis) | 5580 | 9026 | 16911 | 3779 | 5925 | 11681 | 1984 | 3490 | 8374 | 4209 | 6292 | 12438 | 5084 | 7043 | 13046 | 4033 | 6302 | 12281 | 4553 | 6688 | 13100 |
| Bronchiolitis | 6263 | 2747 | 1048 | 5612 | 2403 | 948 | 2142 | 1408 | 1005 | 5102 | 2387 | 1307 | 7023 | 3167 | 1575 | 3109 | 2438 | 1258 | 2634 | 1949 | 1299 |
| **Admissions from E**D |  |  |  |  |  |  |  |  |  |  |  |  |  |  |  |  |  |  |  |  |  |
| All Diagnoses | 21558 | 4046 | 7540 | 20771 | 3088 | 5503 | 18862 | 2596 | 5257 | 20751 | 3185 | 6190 | 22396 | 4124 | 7239 | 14890 | 2747 | 5503 | 20378 | 3230 | 6456 |
| Respiratory Diagnoses (w/o Bronchiolitis) | 476 | 748 | 1368 | 367 | 459 | 976 | 140 | 188 | 674 | 385 | 451 | 1183 | 527 | 703 | 1435 | 349 | 522 | 1191 | 418 | 472 | 1228 |
| Bronchiolitis | 2204 | 443 | 209 | 1883 | 402 | 225 | 716 | 275 | 243 | 1621 | 442 | 334 | 2441 | 614 | 372 | 881 | 456 | 281 | 782 | 303 | 290 |
| **Rome** |  |  |  |  |  |  |  |  |  |  |  |  |  |  |  |  |  |  |  |  |  |
| **Attendances to ED** |  |  |  |  |  |  |  |  |  |  |  |  |  |  |  |  |  |  |  |  |  |
| All Diagnoses | 1412 | 1087 | 2260 | 1226 | 960 | 1893 | 722 | 388 | 734 | 1001 | 755 | 1548 | 1292 | 922 | 1767 | 1262 | 986 | 1685 | 1246 | 947 | 1675 |
| Respiratory Diagnoses (w/o Bronchiolitis) | 154 | 186 | 443 | 140 | 199 | 309 | 26 | 17 | 52 | 102 | 96 | 204 | 108 | 134 | 293 | 157 | 143 | 249 | 185 | 185 | 307 |
| Bronchiolitis | 149 | 62 | 21 | 113 | 49 | 12 | 5 | 5 | 3 | 134 | 56 | 31 | 219 | 68 | 23 | 173 | 91 | 17 | 170 | 64 | 9 |
| **Admissions from ED** |  |  |  |  |  |  |  |  |  |  |  |  |  |  |  |  |  |  |  |  |  |
| All Diagnoses | 388 | 75 | 140 | 328 | 66 | 106 | 283 | 52 | 75 | 289 | 65 | 111 | 337 | 57 | 110 | 343 | 75 | 111 | 310 | 53 | 99 |
| Respiratory Diagnoses (not including Bronchiolitis) | 6 | 6 | 23 | 5 | 6 | 9 | 2 | 1 | 2 | 6 | 4 | 7 | 6 | 2 | 17 | 3 | 5 | 9 | 4 | 3 | 8 |
| Bronchiolitis | 50 | 11 | 4 | 37 | 10 | 6 | 2 | 2 | 0 | 47 | 14 | 10 | 74 | 9 | 6 | 57 | 18 | 6 | 54 | 16 | 5 |
| **Bristol** |  |  |  |  |  |  |  |  |  |  |  |  |  |  |  |  |  |  |  |  |  |
| **Attendances to ED** |  |  |  |  |  |  |  |  |  |  |  |  |  |  |  |  |  |  |  |  |  |
| All Diagnoses | 4965 | 3782 | 7104 | 4763 | 3786 | 6491 | 3312 | 2013 | 3677 | 4965 | 3731 | 7202 | 4992 | 3867 | 6670 | 5148 | 3842 | 6301 | 5095 | 3996 | 6667 |
| Respiratory Diagnoses (w/o Bronchiolitis) | 556 | 1037 | 2707 | 517 | 1100 | 2632 | 169 | 331 | 749 | 501 | 1095 | 3109 | 506 | 1115 | 2679 | 571 | 1038 | 2378 | 666 | 1164 | 2731 |
| Bronchiolitis | 1090 | 606 | 181 | 1065 | 643 | 102 | 116 | 98 | 31 | 1111 | 671 | 163 | 1453 | 754 | 249 | 1318 | 713 | 251 | 1224 | 743 | 319 |
| **Admissions from ED** |  |  |  |  |  |  |  |  |  |  |  |  |  |  |  |  |  |  |  |  |  |
| All Diagnoses | 972 | 383 | 686 | 995 | 406 | 654 | 634 | 208 | 384 | 907 | 359 | 602 | 962 | 366 | 575 | 991 | 363 | 576 | 942 | 425 | 651 |
| Respiratory Diagnoses (w/o Bronchiolitis) | 37 | 54 | 273 | 46 | 58 | 301 | 19 | 7 | 81 | 40 | 48 | 247 | 43 | 45 | 182 | 51 | 44 | 220 | 72 | 50 | 243 |
| Bronchiolitis | 312 | 124 | 51 | 313 | 168 | 26 | 23 | 32 | 11 | 234 | 133 | 32 | 322 | 149 | 64 | 321 | 154 | 55 | 264 | 171 | 101 |
| **Leicester** |  |  |  |  |  |  |  |  |  |  |  |  |  |  |  |  |  |  |  |  |  |
| **Attendances to ED** |  |  |  |  |  |  |  |  |  |  |  |  |  |  |  |  |  |  |  |  |  |
| All Diagnoses | 5175 | 3999 | 7375 | 6068 | 4249 | 7267 | 4154 | 2373 | 3998 | 5942 | 4230 | 8082 | 6201 | 4237 | 6776 | 6217 | 4012 | 6739 | 6411 | 4306 | 7286 |
| Respiratory Diagnoses (w/o Bronchiolitis) | 715 | 1258 | 2751 | 724 | 1376 | 2756 | 296 | 637 | 1013 | 698 | 1365 | 3255 | 705 | 1242 | 2470 | 650 | 1102 | 2208 | 818 | 1229 | 2638 |
| Bronchiolitis | 997 | 578 | 116 | 1030 | 571 | 75 | 172 | 157 | 34 | 998 | 665 | 181 | 1402 | 716 | 150 | 1308 | 670 | 155 | 1103 | 697 | 226 |
| **Admissions from ED** |  |  |  |  |  |  |  |  |  |  |  |  |  |  |  |  |  |  |  |  |  |
| All Diagnoses | 1539 | 630 | 1288 | 1757 | 581 | 1247 | 970 | 184 | 347 | 1075 | 269 | 675 | 1089 | 363 | 489 | 1258 | 347 | 532 | 929 | 284 | 457 |
| Respiratory Diagnoses (w/o Bronchiolitis) | 94 | 184 | 744 | 106 | 143 | 710 | 21 | 31 | 145 | 33 | 48 | 368 | 30 | 55 | 235 | 57 | 48 | 229 | 42 | 36 | 188 |
| Bronchiolitis | 416 | 174 | 36 | 381 | 163 | 18 | 26 | 24 | 6 | 201 | 93 | 26 | 311 | 148 | 17 | 315 | 124 | 35 | 179 | 93 | 23 |
| **Glasgow** |  |  |  |  |  |  |  |  |  |  |  |  |  |  |  |  |  |  |  |  |  |
| **Attendances to ED** |  |  |  |  |  |  |  |  |  |  |  |  |  |  |  |  |  |  |  |  |  |
| All Diagnoses | 6803 | 5243 | 10104 | 7027 | 5511 | 9819 | 4144 | 2683 | 5392 | 6635 | 4980 | 9838 | 7462 | 6064 | 10310 | 7657 | 5394 | 9622 | 7815 | 5940 | 10097 |
| Respiratory Diagnoses (w/o Bronchiolitis) | 493 | 801 | 1838 | 509 | 768 | 1695 | 98 | 230 | 419 | 407 | 567 | 1624 | 452 | 742 | 1502 | 437 | 614 | 1396 | 581 | 744 | 1429 |
| Bronchiolitis | 1012 | 797 | 476 | 999 | 806 | 461 | 66 | 113 | 66 | 837 | 648 | 506 | 1216 | 993 | 389 | 1041 | 796 | 423 | 1106 | 950 | 467 |
| **Admissions from ED** |  |  |  |  |  |  |  |  |  |  |  |  |  |  |  |  |  |  |  |  |  |
| All Diagnoses | 2599 | 1074 | 2003 | 3093 | 1290 | 2367 | 2119 | 693 | 1103 | 2767 | 993 | 2089 | 3028 | 1320 | 2202 | 3079 | 1077 | 1943 | 2933 | 1173 | 1980 |
| Respiratory Diagnoses (not including Bronchiolitis) | 74 | 88 | 447 | 118 | 90 | 493 | 22 | 26 | 117 | 82 | 60 | 484 | 78 | 85 | 390 | 79 | 62 | 372 | 122 | 93 | 392 |
| Bronchiolitis | 342 | 216 | 164 | 422 | 272 | 196 | 20 | 53 | 34 | 285 | 178 | 161 | 414 | 283 | 126 | 365 | 206 | 133 | 344 | 203 | 155 |
| **Edinburgh** |  |  |  |  |  |  |  |  |  |  |  |  |  |  |  |  |  |  |  |  |  |
| **Attendances to ED** |  |  |  |  |  |  |  |  |  |  |  |  |  |  |  |  |  |  |  |  |  |
| All Diagnoses | 5041 | 3881 | 7358 | 4860 | 3766 | 7001 | 3748 | 2125 | 4110 | 5014 | 3516 | 7348 | 5045 | 4067 | 7206 | 4842 | 3570 | 6590 | 5016 | 3712 | 6416 |
| Respiratory Diagnoses (w/o Bronchiolitis) | 387 | 671 | 1565 | 293 | 623 | 1639 | 124 | 260 | 498 | 296 | 646 | 1937 | 331 | 724 | 1831 | 313 | 576 | 1544 | 331 | 691 | 1634 |
| Bronchiolitis | 1042 | 696 | 395 | 1052 | 693 | 350 | 262 | 92 | 69 | 1227 | 555 | 351 | 1417 | 788 | 347 | 1280 | 677 | 327 | 1154 | 677 | 351 |
| **Admissions from ED** |  |  |  |  |  |  |  |  |  |  |  |  |  |  |  |  |  |  |  |  |  |
| All Diagnoses | 1370 | 522 | 980 | 1428 | 508 | 957 | 931 | 219 | 402 | 1252 | 414 | 954 | 1523 | 544 | 1003 | 1517 | 484 | 922 | 1234 | 465 | 761 |
| Respiratory Diagnoses (w/o Bronchiolitis) | 97 | 73 | 298 | 48 | 47 | 319 | 27 | 17 | 81 | 40 | 57 | 391 | 49 | 59 | 405 | 42 | 66 | 359 | 39 | 62 | 306 |
| Bronchiolitis | 402 | 194 | 172 | 472 | 191 | 155 | 89 | 24 | 24 | 427 | 160 | 140 | 585 | 254 | 174 | 548 | 215 | 152 | 354 | 186 | 120 |
| **Iceland** |  |  |  |  |  |  |  |  |  |  |  |  |  |  |  |  |  |  |  |  |  |
| **Attendances to ED** |  |  |  |  |  |  |  |  |  |  |  |  |  |  |  |  |  |  |  |  |  |
| All Diagnoses | 2137 | 1298 | 2391 | 2375 | 1336 | 2351 | 2005 | 1211 | 1979 | 3378 | 1744 | 3508 | 3418 | 1680 | 3497 | 3079 | 1559 | 2620 | 3346 | 1450 | 2966 |
| Respiratory Diagnoses (w/o Bronchiolitis) | 422 | 255 | 627 | 500 | 315 | 611 | 237 | 146 | 348 | 641 | 328 | 764 | 791 | 419 | 992 | 681 | 376 | 654 | 728 | 332 | 639 |
| Bronchiolitis | 72 | 42 | 85 | 87 | 34 | 55 | 29 | 20 | 44 | 99 | 40 | 103 | 170 | 83 | 125 | 140 | 61 | 83 | 170 | 46 | 78 |
| **Admissions from ED** |  |  |  |  |  |  |  |  |  |  |  |  |  |  |  |  |  |  |  |  |  |
| All Diagnoses | 204 | 68 | 123 | 241 | 52 | 170 | 190 | 75 | 103 | 245 | 98 | 168 | 232 | 84 | 228 | 219 | 89 | 139 | 242 | 78 | 171 |
| Respiratory Diagnoses (w/o Bronchiolitis) | 56 | 16 | 46 | 73 | 19 | 51 | 27 | 10 | 22 | 68 | 26 | 66 | 93 | 31 | 125 | 65 | 31 | 59 | 82 | 28 | 56 |
| Bronchiolitis | 38 | 11 | 20 | 33 | 9 | 10 | 12 | 3 | 9 | 29 | 11 | 32 | 47 | 19 | 53 | 40 | 12 | 24 | 58 | 11 | 24 |
| **Romania** |  |  |  |  |  |  |  |  |  |  |  |  |  |  |  |  |  |  |  |  |  |
| **Attendances to ED** |  |  |  |  |  |  |  |  |  |  |  |  |  |  |  |  |  |  |  |  |  |
| All Diagnoses | 2303 | 3175 | 6579 | 2331 | 3239 | 7039 | 1514 | 1510 | 2521 | 2983 | 3649 | 7427 | 3170 | 4279 | 8204 | 2711 | 3906 | 7570 | 2699 | 3308 | 5691 |
| Respiratory Diagnoses (not including Bronchiolitis) | 1253 | 1212 | 1063 | 1695 | 1924 | 1081 | 435 | 452 | 155 | 1819 | 1797 | 845 | 2091 | 2192 | 1085 | 1857 | 2064 | 1106 | 1426 | 1516 | 911 |
| Bronchiolitis | 893 | 559 | 432 | 725 | 458 | 451 | 87 | 60 | 82 | 931 | 616 | 553 | 996 | 509 | 502 | 790 | 504 | 553 | 762 | 426 | 484 |
| **Admissions from ED** |  |  |  |  |  |  |  |  |  |  |  |  |  |  |  |  |  |  |  |  |  |
| All Diagnoses | 1912 | 1061 | 1191 | 1818 | 1107 | 977 | 235 | 112 | 176 | 1987 | 2079 | 814 | 2027 | 1257 | 1089 | 1901 | 1082 | 1011 | 1489 | 710 | 840 |
| Respiratory Diagnoses (not including Bronchiolitis) | 521 | 252 | 250 | 447 | 226 | 196 | 58 | 26 | 45 | 488 | 171 | 123 | 546 | 188 | 176 | 487 | 220 | 223 | 301 | 107 | 149 |
| Bronchiolitis | 500 | 221 | 147 | 452 | 204 | 120 | 43 | 17 | 19 | 513 | 144 | 113 | 548 | 170 | 124 | 483 | 204 | 144 | 436 | 164 | 105 |

**Supplementary Table 3. Summary of attendances and admissions, based on Supplementary Table 2.** Total attendances and admissions by age, study site and diagnosis for Catalonia, Bristol, Leicester, Rome, Glasgow, Edinburgh, Iceland, and Romania, for infants age 0-23 months of age.

| **Region** | **2018-2019** | **2019-2020** | **2020-2021** | **2021-2022** | **2022-2023** | **2023-2025** | **2024-2025** | **STUDY TOTAL** |
| --- | --- | --- | --- | --- | --- | --- | --- | --- |
| **Catalonia** |  |  |  |  |  |  |  |  |
| **Attendances to ED** | 230,688 | 175,741 | 154,435 | 201,998 | 209,250 | 182,899 | 202,275 | 1,357,286 |
| **Admissions from ED** | 43,116 | 41,542 | 37,724 | 41,502 | 44,792 | 29,780 | 40,756 | 279,212 |
| **Bristol** |  |  |  |  |  |  |  |  |
| **Attendances to ED** | 15,851 | 15,040 | 9,002 | 15,898 | 15,529 | 15,291 | 15,758 | 102,369 |
| **Admissions from ED** | 1,944 | 1,990 | 1,268 | 1,814 | 1,924 | 1,982 | 1,884 | 12,806 |
| **Leicester** |  |  |  |  |  |  |  |  |
| **Attendances to ED** | 16,549 | 17,584 | 10,525 | 18,254 | 17,214 | 16,968 | 18,003 | 115,097 |
| **Admissions from ED** | 3,078 | 3,514 | 1,940 | 2,150 | 2,178 | 2,516 | 1,858 | 17,234 |
| **Glasgow** |  |  |  |  |  |  |  |  |
| **Attendances to ED** | 22,150 | 22,357 | 12,219 | 21,453 | 23,836 | 22,673 | 23,852 | 148,540 |
| **Admissions from ED** | 5,198 | 6,186 | 4,238 | 5,534 | 6,056 | 6,158 | 5,866 | 39,236 |
| **Edinburgh** |  |  |  |  |  |  |  |  |
| **Attendances to ED** | 16,280 | 15,627 | 9,983 | 15,878 | 16,318 | 15,002 | 15,144 | 104,232 |
| **Admissions from ED** | 2,740 | 2,856 | 1,862 | 2,504 | 3,046 | 3,034 | 2,468 | 18,510 |
| **UK Total** |  |  |  |  |  |  |  |  |
| **Attendances to ED** | 70,830 | 70,608 | 41,729 | 71,483 | 72,897 | 69,934 | 72,757 | 470,238 |
| **Admissions from ED** | 12,960 | 14,546 | 9,308 | 12,002 | 13,204 | 13,690 | 12,076 | 87,786 |
| **Rome** |  |  |  |  |  |  |  |  |
| **Attendances to ED** | 4,759 | 4,079 | 1,844 | 3,304 | 3,981 | 3,933 | 3,868 | 25,768 |
| **Admissions from ED** | 776 | 656 | 566 | 578 | 674 | 686 | 620 | 4,556 |
| **Iceland** |  |  |  |  |  |  |  |  |
| **Attendances to ED** | 5,826 | 6,062 | 5,195 | 8,630 | 8,595 | 7,258 | 7,762 | 49,328 |
| **Admissions from ED** | 408 | 482 | 380 | 490 | 464 | 438 | 484 | 3,146 |
| **Romania** |  |  |  |  |  |  |  |  |
| **Attendances to ED** | 12,057 | 12,609 | 5,545 | 14,059 | 15,653 | 14,187 | 11,698 | 85,808 |
| **Admissions from ED** | 3,824 | 3,636 | 470 | 3,974 | 4,054 | 3,802 | 2,978 | 22,738 |

**Supplementary Table 4. Risk ratios for bronchiolitis attendances to Emergency Departments (EDs), comparing grouped data from preceding seasons to the 2024/2025 season.** Risk ratios with confidence interval (CI) 95% obtained with a Poisson regression for attendances to the ED with a primary diagnosis of bronchiolitis in the 2024-2025 season compared to grouped “pre-nirse” 2018-2019, 2019-2020, 2021-2022 and 2022-2023 seasons, for study sites. Results with ^†^ are those including 2023-2024 as a reference year. P-values with * are those of less than 0.05 (95% significance).

| ***Location*** | ***Year*** | **Age Group** | **RR (95% CI)** | **P-value** |
| --- | --- | --- | --- | --- |
| ***Catalonia*** | ***2024-2025*** | **<6m** | **0.45 (0.43-0.47)** | **< 0.0001*** |
|  |  | **6-11m** | **0.72 (0.69-0.76)** | **< 0.0001*** |
|  |  | **12-23m** | **1.08 (1.01-1.14)** | **0.0182*** |
|  | ***2024-2025***^†^ | **<6m** | **0.49 (0.48-0.52)** | **< 0.0001*** |
|  |  | **6-11m** | **0.73 (0.70-0.77)** | **< 0.0001*** |
|  |  | **12-23m** | 1.06 (0.99-1.12) | 0.0698 |
| ***Rome*** | ***2024-2025*** | **<6m** | 1.09 (0.92-1.30) | 0.3002 |
|  |  | **6-11m** | 1.07 (0.81-1.41) | 0.6268 |
|  |  | **12-23m** | **0.46 (0.23-0.92)** | **0.0271*** |
|  | ***2024-2025***^†^ | **<6m** | 1.07 (0.91-1.27) | 0.4093 |
|  |  | **6-11m** | 0.98 (0.75-1.28) | 0.8614 |
|  |  | **12-23m** | **0.47 (0.24-0.93)** | **0.0311*** |
| ***Bristol*** | ***2024-2025*** | **<6m** | 1.00 (0.94-1.07) | 0.9472 |
|  |  | **6-11m** | 1.05 (0.97-1.14) | 0.2002 |
|  |  | **12-23m** | **1.89 (1.66-2.16)** | **< 0.0001*** |
|  | ***2024-2025***^†^ | **<6m** | 0.99 (0.93-1.05) | 0.7050 |
|  |  | **6-11m** | 1.04 (0.96-1.13) | 0.2934 |
|  |  | **12-23m** | **1.71 (1.50-1.94)** | **< 0.0001*** |
| ***Leicester*** | ***2024-2025*** | **<6m** | **0.91 (0.85-0.97)** | **0.0045*** |
|  |  | **6-11m** | 1.07 (0.98-1.16) | 0.1167 |
|  |  | **12-23m** | **1.75 (1.50-2.05)** | **< 0.0001*** |
|  | ***2024-2025***^†^ | **<6m** | **0.89 (0.83-0.95)** | **0.0003*** |
|  |  | **6-11m** | 1.05 (0.97-1.14) | 0.2577 |
|  |  | **12-23m** | **1.66 (1.43-1.93)** | **< 0.0001*** |
| ***Glasgow*** | ***2024-2025*** | **<6m** | 0.97 (0.91-1.04) | 0.4112 |
|  |  | **6-11m** | 1.07 (1.00-1.16) | 0.0509 |
|  |  | **12-23m** | 1.01 (0.91-1.12) | 0.8232 |
|  | ***2024-2025***^†^ | **<6m** | 0.99 (0.92-1.05) | 0.6813 |
|  |  | **6-11m** | **1.08 (1.00-1.16)** | **0.0410*** |
|  |  | **12-23m** | 1.02 (0.92-1.13) | 0.7079 |
| ***Edinburgh*** | ***2024-2025*** | **<6m** | 0.97 (0.91-1.03) | 0.3406 |
|  |  | **6-11m** | 1.02 (0.93-1.11) | 0.6994 |
|  |  | **12-23m** | 1.10 (0.98-1.23) | 0.1229 |
|  | ***2024-2025***^†^ | **<6m** | 0.95 (0.89-1.01) | 0.0976 |
|  |  | **6-11m** | 1.01 (0.93-1.09) | 0.8907 |
|  |  | **12-23m** | 1.10 (0.98-1.23) | 0.1119 |
| ***Iceland*** | ***2024-2025*** | **<6m** | **1.34 (1.12-1.60)** | **0.0012*** |
|  |  | **6-11m** | 0.97 (0.70-1.33) | 0.8313 |
|  |  | **12-23m** | 0.84 (0.66-1.07) | 0.1604 |
|  | ***2024-2025***^†^ | **<6m** | **1.29 (1.08-1.53)** | **0.0039*** |
|  |  | **6-11m** | 0.93 (0.68-1.27) | 0.6471 |
|  |  | **12-23m** | 0.84 (0.66-1.07) | 0.1488 |
| ***Romania*** | ***2024-2025*** | **<6m** | **0.86 (0.79-0.93)** | **0.0001*** |
|  |  | **6-11m** | **0.86 (0.77-0.96)** | **0.0052*** |
|  |  | **12-23m** | **1.28 (1.16-1.42)** | **< 0.0001*** |
|  | ***2024-2025***^†^ | **<6m** | **0.88 (0.81-0.95)** | **0.0010*** |
|  |  | **6-11m** | **0.89 (0.80-0.98)** | **0.0230*** |
|  |  | **12-23m** | **1.26 (1.14-1.39)** | **< 0.0001*** |

**Supplementary** **Table 5. Risk ratios for bronchiolitis admissions from Emergency Departments (EDs), comparing grouped data from preceding seasons to 2023-2024.** Risk ratios with confidence interval (CI) 95% obtained with a Poisson regression for attendances to the ED with a primary diagnosis of bronchiolitis in the 2024-2025 season compared to grouped “pre-nirse” 2018-2019, 2019-2020, 2021-2022 and 2022-2023 seasons, for study sites. Results with ^†^ are those including 2023-2024 as a reference year. P-values with * are those of less than 0.05 (95% significance).

| ***Location*** | ***Year*** | **Age Group** | **RR (95% CI)** | **P-value** |
| --- | --- | --- | --- | --- |
| ***Catalonia*** | ***2024-2025*** | **<6m** | **0.40 (0.37-0.43)** | **< 0.0001*** |
|  |  | **6-11m** | **0.71 (0.63-0.80)** | **< 0.0001*** |
|  |  | **12-23m** | 1.04 (0.92-1.19) | 0.5213 |
|  | ***2024-2025***^†^ | **<6m** | 0.44 (0.41-0.48) | **< 0.0001*** |
|  |  | **6-11m** | 0.70 (0.62-0.79) | **< 0.0001*** |
|  |  | **12-23m** | 1.04 (0.91-1.18) | 0.5844 |
| ***Rome*** | ***2024-2025*** | **<6m** | 1.12 (0.83-1.52) | 0.4445 |
|  |  | **6-11m** | **1.80 (1.02-3.20)** | **0.0432*** |
|  |  | **12-23m** | 0.91 (0.35-2.36) | 0.8418 |
|  | ***2024-2025***^†^ | **<6m** | 1.11 (0.83-1.48) | 0.4936 |
|  |  | **6-11m** | 1.65 (0.95-2.85) | 0.0756 |
|  |  | **12-23m** | 0.91 (0.36-2.34) | 0.8485 |
| ***Bristol*** | ***2024-2025*** | **<6m** | 0.91 (0.80-1.04) | 0.1674 |
|  |  | **6-11m** | 1.06 (0.89-1.26) | 0.4950 |
|  |  | **12-23m** | **2.26 (1.77-2.89)** | **<0.0001*** |
|  | ***2024-2025***^†^ | **<6m** | 0.90 (0.79-1.03) | 0.1169 |
|  |  | 6-11m | 1.04 (0.88-1.23) | 0.6658 |
|  |  | **12-23m** | **2.10 (1.67-2.66)** | **<0.0001*** |
| ***Leicester*** | ***2024-2025*** | **<6m** | **0.80 (0.69-0.94)** | **0.0061*** |
|  |  | **6-11m** | 1.04 (0.84-1.30) | 0.7098 |
|  |  | **12-23m** | **1.91 (1.21-3.01)** | **0.0053*** |
|  | ***2024-2025***^†^ | **<6m** | **0.80 (0.69-0.94)** | **0.0056*** |
|  |  | **6-11m** | 1.03 (0.83-1.28) | 0.8027 |
|  |  | **12-23m** | **1.59 (1.02-2.47)** | **0.0416*** |
| ***Glasgow*** | ***2024-2025*** | **<6m** | 0.92 (0.82-1.04) | 0.1690 |
|  |  | **6-11m** | **0.85 (0.73-0.99)** | **0.0396*** |
|  |  | **12-23m** | 1.05 (0.88-1.25) | 0.6006 |
|  | ***2024-2025***^†^ | **<6m** | 0.93 (0.83-1.05) | 0.2495 |
|  |  | **6-11m** | 0.86 (0.74-1.00) | 0.0513 |
|  |  | **12-23m** | 1.06 (0.90-1.26) | 0.4789 |
| ***Edinburgh*** | ***2024-2025*** | **<6m** | **0.85 (0.76-0.95)** | **0.0043*** |
|  |  | **6-11m** | 1.00 (0.85-1.17) | 0.9533 |
|  |  | **12-23m** | 0.96 (0.79-1.16) | 0.6657 |
|  | ***2024-2025***^†^ | **<6m** | **0.84 (0.75-0.93)** | **0.0015*** |
|  |  | **6-11m** | 0.98 (0.83-1.14) | 0.7524 |
|  |  | **12-23m** | 0.96 (0.79-1.16) | 0.6587 |
| ***Iceland*** | ***2024-2025*** | **<6m** | **1.50 (1.11-2.04)** | **0.0086*** |
|  |  | **6-11m** | 0.85 (0.44-1.64) | 0.6300 |
|  |  | **12-23m** | 0.84 (0.54-1.31) | 0.4400 |
|  | ***2024-2025***^†^ | **<6m** | **1.46 (1.09-1.96)** | **0.0114*** |
|  |  | **6-11m** | 0.89 (0.47-1.69) | 0.7201 |
|  |  | **12-23m** | 0.84 (0.54-1.29) | 0.4179 |
| ***Romania*** | ***2024-2025*** | **<6m** | **1.13 (1.02-1.25)** | **0.0242*** |
|  |  | **6-11m** | **1.72 (1.45-2.04)** | **<0.0001*** |
|  |  | **12-23m** | 1.01 (0.82-1.25) | 0.9285 |
|  | ***2024-2025***^†^ | **<6m** | **1.13 (1.02-1.25)** | **0.0173*** |
|  |  | **6-11m** | **1.61 (1.37-1.90)** | **<0.0001*** |
|  |  | **12-23m** | 0.98 (0.80-1.20) | 0.8502 |

## 2. Supplementary Figures


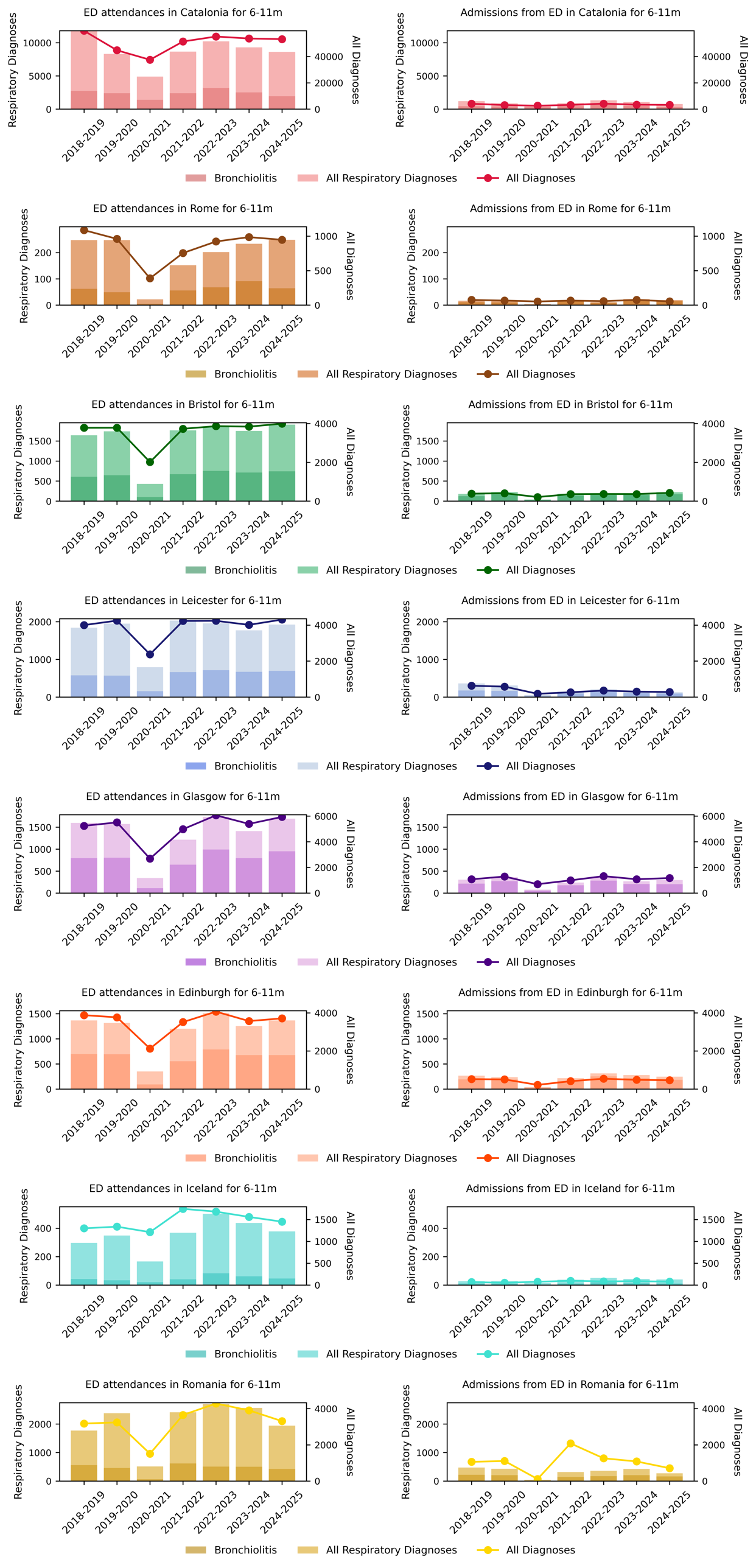


**Figure S1. Emergency Department (ED) attendances and hospitalisations for infants 6-11 months of age at study sites.** The number of bronchiolitis (dark) and all respiratory diagnoses (light) cases attending each site’s ED and admitted from the ED are shown on the right and left, respectively, with numbers shown on the left t y-axis. The right y-axis shows the total number of ED attendances and admissions, respectively at the study sites.





**Figure S2. Emergency Department (ED) attendances and hospitalisations for infants 12-23 months of age at study sites.** The number of bronchiolitis (dark) and all respiratory diagnoses (light) cases attending each site’s ED and admitted from the ED are shown on the right and left, respectively, with numbers shown on the left t y-axis. The right y-axis shows the total number of ED attendances and admissions, respectively at the study sites.

## 3. STROBE checklist

|  | **Item No** | **Recommendation** |
| --- | --- | --- |
| **Title and abstract** | 1 | (*a*) Indicate the study’s design with a commonly used term in the title or the abstract: **multinational retrospective data analysis** |
|  |  | (*b*) Provide in the abstract an informative and balanced summary of what was done and what was found: **this information is included in the methods section of the abstract, “Bronchiolitis diagnoses in the 2024-2025 season were compared to previous pre-intervention seasons (2018-2023, excluding the 2020-2021 COVID-19 year) by applying a generalised linear model in Poisson regression to obtain risk ratios (RR) and 95% confidence intervals (95%CI).”** |
| **Introduction** | | |
| Background/rationale | 2 | Explain the scientific background and rationale for the investigation being reported: **lines 126 to 149** |
| Objectives | 3 | State specific objectives, including any prespecified hypotheses: **lines 149-156** |
| **Methods** | | |
| Study design | 4 | Present key elements of study design early in the paper: **lines 159-168** |
| Setting | 5 | Describe the setting, locations, and relevant dates, including periods of recruitment, exposure, follow-up, and data collection: **settings, locations, and relevant dates are described between lines 159 and 272; periods of recruitment and data collection in lines 247 to 266.** |
| Participants | 6 | (*a*) *Cohort study*—Give the eligibility criteria, and the sources and methods of selection of participants. Describe methods of follow-up.  *Case-control study*—Give the eligibility criteria, and the sources and methods of case ascertainment and control selection. Give the rationale for the choice of cases and controls.  *Cross-sectional study*—Give the eligibility criteria, and the sources and methods of selection of participants: **lines 248-252.** |
|  |  | (*b*) *Cohort study*—For matched studies, give matching criteria and number of exposed and unexposed  *Case-control study*—For matched studies, give matching criteria and the number of controls per case |
| Variables | 7 | Clearly define all outcomes, exposures, predictors, potential confounders, and effect modifiers. Give diagnostic criteria, if applicable: **lines 256 to 294** |
| Data sources/ measurement | 8* | For each variable of interest, give sources of data and details of methods of assessment (measurement). Describe comparability of assessment methods if there is more than one group: **lines 228 to 294** |
| Bias | 9 | Describe any efforts to address potential sources of bias: **lines 273 to 279** |
| Study size | 10 | Explain how the study size was arrived at**: sample size was directly related to the available data on ED attendances and hospital admissions for all the participating sites (lines 248 to 252).** |
| Quantitative variables | 11 | Explain how quantitative variables were handled in the analyses. If applicable, describe which groupings were chosen and why: **described in lines 275 to 311**. |
| Statistical methods | 12 | (*a*) Describe all statistical methods, including those used to control for confounding |
|  |  | (*b*) Describe any methods used to examine subgroups and interactions |
|  |  | (*c*) Explain how missing data were addressed |
|  |  | (*d*) *Cohort study*—If applicable, explain how loss to follow-up was addressed  *Case-control study*—If applicable, explain how matching of cases and controls was addressed.  *Cross-sectional study*—If applicable, describe analytical methods taking account of sampling strategy |
|  |  | (*e*) Describe any sensitivity analyses **all the statistical analysis are explained between lines 275 and 311.** |
| **Results** | | |
| Participants | 13* | (a) Report numbers of individuals at each stage of study—eg numbers potentially eligible, examined for eligibility, confirmed eligible, included in the study, completing follow-up, and analysed: **We collected data on individuals attending ED and admitted to hospital, this is described between lines 248 and 252, including Supplementary Tables 2 and 3.** |
|  |  | (b) Give reasons for non-participation at each stage: **there were no reasons for non-participating in the study because this is a retrospective analysis including aggregated data of all the ED attendances and hospital admissions from the participating sites.** |
|  |  | (c) Consider use of a flow diagram: **not applicable in this study.** |
| Descriptive data | 14* | (a) Give characteristics of study participants (eg demographic, clinical, social) and information on exposures and potential confounders: **not available in this study, we used aggregated data based on the number of ED attendances and hospital admissions for all-causes, respiratory diagnoses and bronchiolitis for different age groups.** |
|  |  | (b) Indicate number of participants with missing data for each variable of interest: **no missing data for variables of interest.** |
|  |  | (c) *Cohort study*—Summarise follow-up time (eg, average and total amount) |
| Outcome data | 15* | *Cohort study*—Report numbers of outcome events or summary measures over time |
|  |  | *Case-control study—*Report numbers in each exposure category, or summary measures of exposure |
|  |  | *Cross-sectional study—*Report numbers of outcome events or summary measures: **lines 268 to 272.** |
| Main results | 16 | (*a*) Give unadjusted estimates and, if applicable, confounder-adjusted estimates and their precision (eg, 95% confidence interval). Make clear which confounders were adjusted for and why they were included |
|  |  | (*b*) Report category boundaries when continuous variables were categorized |
|  |  | (*c*) If relevant, consider translating estimates of relative risk into absolute risk for a meaningful time period: N/A. |
| Other analyses | 17 | Report other analyses done—eg analyses of subgroups and interactions, and sensitivity analyses: **lines 386 to 396.** |
| **Discussion** |  |  |
| Key results | 18 | Summarise key results with reference to study objectives: **lines 399 to 452** |
| Limitations | 19 | Discuss limitations of the study, taking into account sources of potential bias or imprecision. Discuss both direction and magnitude of any potential bias: **lines 484** to **498.** |
| Interpretation | 20 | Give a cautious overall interpretation of results considering objectives, limitations, multiplicity of analyses, results from similar studies, and other relevant evidence: **lines 454** to **481.** |
| Generalisability | 21 | Discuss the generalisability (external validity) of the study results: **lines 465** to **470.** |
| **Other information** |  |  |
| Funding | 22 | Give the source of funding and the role of the funders for the present study and, if applicable, for the original study on which the present article is based**: lines 329**. |

C

*Give information separately for cases and controls in case-control studies and, if applicable, for exposed and unexposed groups in cohort and cross-sectional studies.
